# Metastatic HPV‐related oropharyngeal carcinoma cured with chemoradiotherapy: importance of pretherapy biomolecular assessment

**DOI:** 10.1002/ccr3.1107

**Published:** 2017-11-24

**Authors:** Francesco Perri, Francesco Longo, Giuseppina Della Vittoria Scarpati, Salvatore Pisconti, Vito Longo, Raffaele Addeo, Fabio Carducci, Carlo Buonerba, Franco Fulciniti, Raffaele Solla

**Affiliations:** ^1^ Medical Oncology Department POC SS Annunziata Taranto Italy; ^2^ Otolaryngology Unit National Tumour Institute of Naples, G Pascale Naples Italy; ^3^ Department of Medical Oncology Hospital S Giovanni di Dio Naples Italy; ^4^ Otolaryngology Unit POC SS Annunziata Taranto Italy; ^5^ Medical Oncology Department University Federico II of Naples Naples Italy; ^6^ Pathology Unit Clinica Luganese Lugano Switzerland; ^7^ Institute of Biostructure and Bioimaging National Council of Research Naples Italy; ^8^ Department of Advanced Biomedical Sciences Federico II University School of Medicine Naples Italy

**Keywords:** Cyclin D1, human papillomavirus, oropharyngeal carcinoma, P16, p53

## Abstract

Pretherapy assessment has a crucial role in the management of advanced oropharyngeal carcinoma. The case report represents an example of how translational research may help to optimize the therapeutic options and to choose a well‐shaped therapy adapted to the tumor and the patient.

## Introduction

Oropharyngeal carcinomas (OCs) account for 20% of squamous cell carcinomas of the head and neck (SCCHN), and their incidence is steadily increasing 1. OCs often are diagnosed as locally advanced (LA) disease, namely staged as T1N1 until T4N3 according to American Joint Committee against Cancer (AJCC) staging system. Locally advanced oropharyngeal carcinomas (LAOCs) are currently managed with concomitant cisplatin and radiation therapy, according to the National Comprehensive Cancer Network (NCCN) guidelines, and upfront surgery is seldom necessary, being recommended only for very advanced T4 lesions 2, 3.

Conservative strategy represents the preferred treatment option for LAOCs, being most of them characterized by good response to both chemo‐ and radiotherapy. Nevertheless, a number of patients treated with chemoradiotherapy often experience recurrent disease and show poor prognosis 4. Progressive disease or early recurrence after chemoradiation may characterize some chemo‐ and radio‐resistant forms, which show poor prognosis and scarce response to conservative treatments 5, 6.

Recently, an increase in diagnosis of some OCs has been documented, especially those arising from tonsil and base of tongue. This phenomenon has been associated with the increased incidence of human papillomavirus (HPV)‐positive tumors 7. HPV‐positive OCs generally show good prognosis and, in some reports, also a better response to chemo‐ and radiotherapy, when compared with the HPV‐negative counterparts 8, 9. This last feature has paved the way to a number of clinical trials aimed to de‐intensify standard treatments, substituting, for example, cisplatin with cetuximab given concomitantly to radiotherapy, or removing definitively surgery from the therapeutic armamentarium. Nevertheless, some HPV‐positive OCs show a grim prognosis and a poor response to chemoradiotherapy, especially if diagnosed in strong smokers or drinkers and in the 6th or 7th decade of age 8, 10, 11. One interpretation is that not all the HPV‐positive tumors are also HPV‐related neoplasms. HPV‐driven cancerogenesis leads to the onset of tumors characterized by special features, such as cell cycle disruption, high proliferating index, and, consequently, high sensitivity to both chemo‐ and radiotherapy. Moreover, HPV‐related tumors often are diagnosed in the young adult, nonsmokers, or slightly smokers, with no anamnesis of alcohol consumption and with history of several sexual partners 12, 13. HPV‐related OCs show p16 and p21 overexpression, TP53 not mutated, CCND1 wild‐type status, and low epidermal growth factor receptor (EGFR) expression at immunostaining. The tobacco‐ and alcohol‐related OCs, namely the HPV‐negative counterpart, often show the opposite features 14, 15, 16, 17.

In the future, it is likely that making a distinction between HPV‐ and non‐HPV‐related OCs will be of utmost importance, being the first very chemo‐ and radiosensitive, and thus more suitable for conservative treatments, even if diagnosed at advanced stage.

## Case Presentation

**Figure 1:**
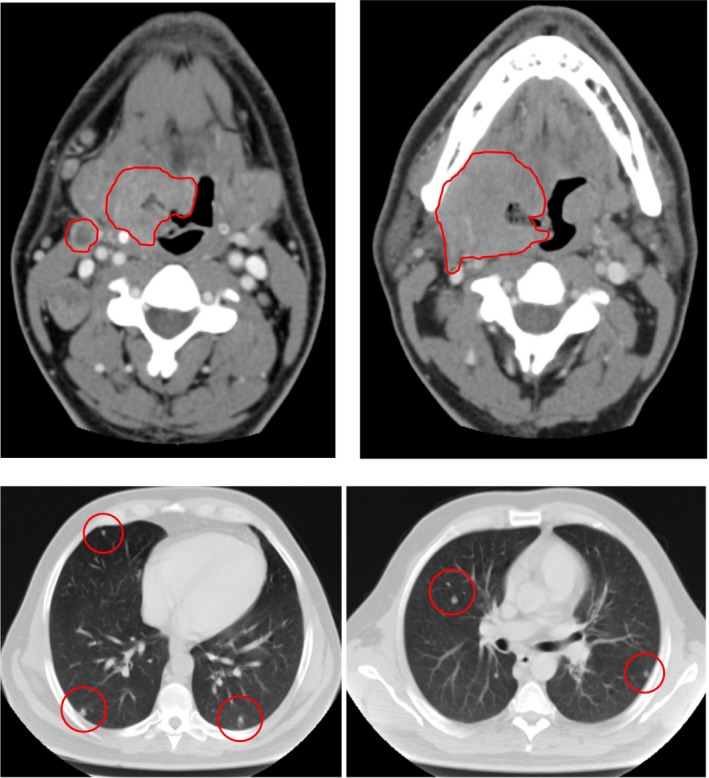
June 2013; first staging with computed tomography.

About 32 months ago, a 39‐year‐old Caucasian male patient came to our attention, suffering from dysphagia and odynophagia which were persistent after several lines of antibiotics therapy. He referred us to be a slight smoker (<10 cigarettes in a month) and did not report alcohol consumption. At a clinical examination, a wide lesion arising from the right tonsil was detected, and at the neck examination, an omolateral laterocervical mass was found interesting the II level according to Robbins classification. A fibroscopy confirmed the presence of a wide exophytic mass arising from the right tonsil and infiltrating the omolateral tonsillar pillar. A biopsy of the lesion was performed during the fibroscopy. Histopathologic examination of the biopsy sample confirmed the clinical suspicion, identifying a poorly differentiated squamous cell carcinoma of the right tonsil. The neoplasm was staged by performing a computed tomography of the head, neck, thorax, and abdomen, which better defined the primary tumor extension. The mass measured 5 centimeters in the biggest diameter and infiltrated the base of tongue and the floor of mouth. A single 2,5 centimeters lymph node metastasis was detected at the station II of the laterocervical chain. Moreover, multiple bilateral lung lesions were detected, the biggest of them being 1,5 centimeters length. A following Positron emission tomography (PET/TC) confirmed the malignant nature of the oropharyngeal lesion, which appeared as an area showing high uptake (SUV 12.6); both the lymph node (SUV: 10) and lung lesions (SUV: 6) also strongly pick up the FDG (fluoro‐deoxy‐glucose) (Fig. 1). Stage of disease was T4N1M1 (IVc according to AJCC).

### Additional determinations

Histopathologic diagnosis was a poorly differentiated squamous cell carcinoma. The HPV of the neoplasm was studied by in situ hybridization with positive result. A number of biological features were studied by immunohistochemistry, such as p16, p21, EGFR, TP53, Akt, and CCND1 status. Immunohistochemistry was employed to detect EGFR, P16, P21, Cyclin D1 (which is the product of CCND1 gene), and phospho‐Akt expression. Polymerase chain reaction (PCR) was used to identify mutations in the TP53 gene. As a result, p16 and p21 were overexpressed, while EGFR, phospho‐Akt, and Cyclin D1 were not. No TP53 mutations were detected.

Immunohistochemistry was performed on representative 4 ‐*μ*m sections cut from formalin‐fixed, paraffin‐embedded tissue blocks, using a monoclonal antibody to p16 (MTM Laboratories; monoclonal; 1:1 dilution) on a Ventana Benchmark LT automated immunostainer (Ventana Medical Systems, Tucson AZ) according to standard protocols. P16‐positive samples have been considered all that displaying a tissue expression of at least 51%.

Primary antibody incubation was carried out overnight at room temperature using a mouse monoclonal antibody for Cyclin D1 (Clone DCS‐6, DAKO Corp. A/S, Glostrup, Denmark) at 1:50 dilution. We graded the distribution of positive cells in tumors that stained for Cyclin D1 as follows: grade 1, focal staining in <10% of tumor cells; grade 2, fairly widespread staining in 10–50% of tumor cells; and grade 3, diffuse staining in more than 50% of tumor cells. We considered as “overexpressed” values above 50%.

Epidermal growth factor receptor protein expression was assessed by IHC with the Dako EGFR PharmDx kit (DakoCytomation, Berkeley, CA). Samples were classified as EGFR IHC overexpressed if ≥50% of the tumor cells demonstrated membranous staining of any intensity.

Detection of TP53 mutations was carried out using the IARC (International Agency for Research on Cancer) database, reporting mutations in several loci included between exons 2 and 11. PCR was employed, and primers located at least 50 bp away from the ends of each exon were designed to amplify exons 1–11 of the TP53 gene.

We have used Phospho‐Akt (Ser473) (736E11) Rabbit mAb to measure PI3K/Akt/mTor pathway activation in paraffin‐embedded tissue samples. The intensity is designated as 0 when no tumor cells stained, 1+ when 10–20% of cells stained (weak), 2+ when 20–50% of cells stained (moderate), and 3+ when >50% of cells stained (strong).

Santa Cruz Biotechnology p21 antibody (Santa, sc‐6246) was used. The intensity of the staining was scored as follows: low when 1–10% of cells showed positive staining, moderate when 11–50% of them showed positive staining, and severe when more than 50% showed positive staining.

### Treatment

Given the disease extension (IVc sec AJCC), a polychemotherapy regimen, based on cisplatin–cetuximab–5‐fluorouracil, was recommended by American and European guidelines. Nevertheless, on the basis of HPV positivity, with wild‐type Cyclin D1, p16, and p21 overexpressed, TP53 wild type and EGFR slightly expressed, we could expect a good response to chemo‐ and radiotherapy. Thus, we chose to employ induction chemotherapy conditionally followed by chemoradiotherapy, in case of complete response or complete disappearance of lung metastases. We chose to use a strongly active scheme, and 3 months after the first diagnosis, three cycles of cisplatin–docetaxel, as induction chemotherapy, were administered. Both cisplatin and docetaxel were employed at a dose of 80 mg/m2 on day one, and they were repeated every 3 weeks. Acute toxicity was mild being grade 2 neutropenia after the third cycle, and grade 3 alopecia and grade 1 nausea after 2 weeks from the start of chemotherapy.

### First restaging

**Figure 2:**
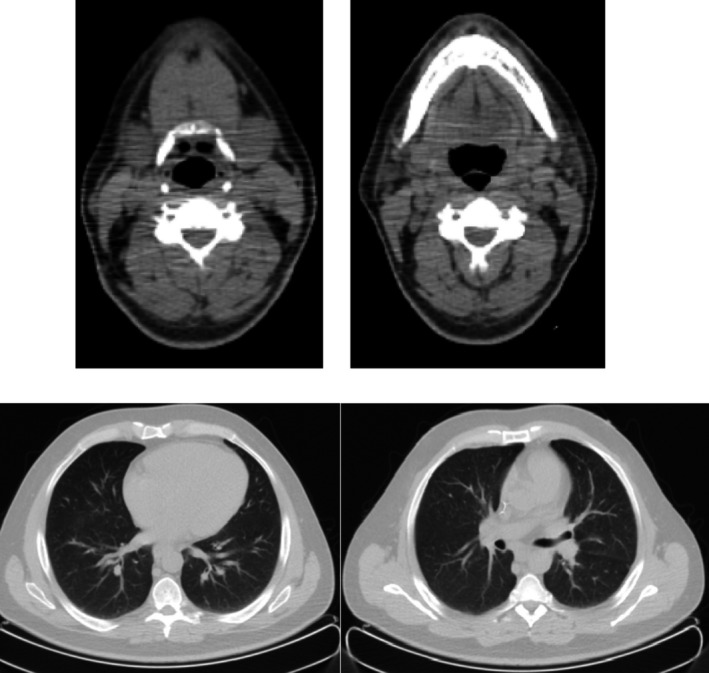
September 2013; first restaging after induction chemotherapy.

Two months after the first chemotherapy cycle, a restaging CT scan was performed, and as a result, complete disappearance of lung lesions as well as the oropharyngeal mass was observed. The lymph node lesion appeared strongly reduced when compared with previous CT scan (Fig. 2). A PET/TC was also performed, and it confirmed the CT scan findings, and as a result, only the oropharyngeal lesion persisted, but its uptake was strongly reduced (SUV 2.2 vs. 12.6).

### Treatment

On the basis of the optimal response obtained after induction chemotherapy, as well as the complete disappearance of distant lesions, we chose to continue the therapeutic program and to administer concurrent chemoradiation. External beam radiotherapy was administered using 6 MV of energy photons of the linear accelerator (LINAC). A 3D conformal technique was employed, and a total dose of 70 Gy was reached using a conventional fractionating regimen (2.00 Gy per fraction). The target to be irradiated was considered to be the primary tumor plus bilateral laterocervical lymph nodes starting from level Ib to level IV. This last level was considered as the CTV (clinical target volume). A margin of 1 centimeter was added to CTV with the aim of avoiding positioning errors and involuntary patient movements, and as a result, a larger volume was obtained, named PTV (planning target volume). More in particular, we delineated a PTV1, which comprised the gross tumor volume (GTV) in the oropharynx plus the bilateral lymph node stations from Ib to IV, and we administered a maximum dose of 60 Gy on this field. Then, we identified a PTV2, which comprised only the oropharyngeal mass and the visible lymph nodal lesion, in the right laterocervical region. We reached a total dose of 66 Gy on PTV2. Finally, we designed a third and shorter treatment volume, namely PTV3, which included only the gross oropharyngeal tumor volume, and we reached the total 70 Gy dose on it. For CTV delineation, we used prechemotherapy tumor extension. Concomitant chemotherapy was performed employing cisplatin at dose of 80 mg for square meter of body surface given every 3 weeks. The aforementioned chemoradiation protocol was administered for a duration of 7 weeks, and it was started 25 days after chemotherapy completion. Acute toxicity was moderate, being grade 3 dysphagia the worse side effect encountered. It appeared after 5 weeks from the start of chemoradiation and lasted throughout the treatment, requiring also the feeding tube apposition. Grade 2 nausea and skin erythema started during the second week of treatment and lasted about 1 month. No further side effects were seen after the completion of the chemoradiation.

### Second restaging

**Figure 3:**
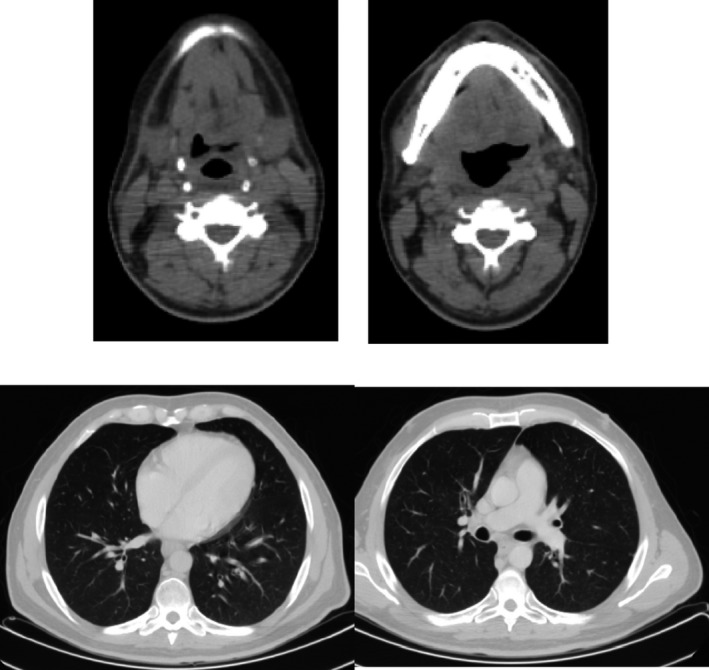
February 2014; second restaging after chemoradiation (CT scan).

One month later, a CT scan was performed, and as a result, only a modest asymmetry of the oropharyngeal cavity was detected, without visible lesions or contrast enhancement. Neither laterocervical norlung masses were seen. Two months later, a PET/TC did not detect any pathologic uptake in the examined body parts explored. In addition, we performed a complete clinical examination and a fibroscopy that did not show suspicious lesions or features. We judged the patient to be in complete response (CR) (Fig. 3), and we addressed him to a follow‐up program.

### Follow‐up

A first follow‐up visit was made 3 months later and consisted in a CT scan and a complete clinical examination. Fibroscopy did not detect suspicious lesions. A following CT scan was performed 4 months later and did not highlight significant features. A following CT scan was performed 6 months later and did not detect pathological features. The last CT scan was performed 6 months later and was considered as negative for disease recurrence.

Actually, the patient is alive, and at a last follow‐up visit, on December 2016, he was recurrence‐free.

## Discussion

Epidemiology of SCCHNs has profoundly changed in the last decade, mainly due to the increasing frequency of virus‐related malignancies 9. HPV infects the upper aerodigestive tract and the proximal airways, showing high tropism for some areas, among oropharynx. OCs, but also other HPV‐related SCCHNs, are characterized by peculiar features that distinguish them significantly from those non‐HPV‐related.

In particular, the cancerogenesis process in head and neck cancer, among oropharyngeal cancer, is mainly due to the mutagens present in tobacco and alcohol, which may alter DNA in specific loci leading to neoplastic progression 18. HPV‐related cancerogenesis is very different, and HPV‐related malignancies are characterized by good prognosis and good response to both chemo‐ and radiotherapy in clinical trials 19, 20. Given their chemo‐ and radiosensitivity, HPV‐related head and neck carcinomas could be treated preferably with conservative approaches such as sequential or concurrent chemoradiotherapy. Nevertheless, it is crucial to distinguish between HPV‐positive and HPV‐related tumors. In fact, some neoplasms which show positivity for HPV are characterized in any case by poor prognosis and scarce response to both chemo‐ and radiotherapy, because they arise in heavy smokers and/or drinkers. In this last case, the impact of HPV on cancerogenesis is only marginal. On the other hand, when HPV is the main promoter of cancerogenesis, tumor cells show typical features, such as high proliferating index, a low number of DNA changes, and high sensitivity to either chemical mutagens or ionizing radiations.

The immunohistochemical panel, which we have chosen to adopt, has led us to identify a tumor strongly related to HPV effect. This type of cancerogenesis is strongly HPV‐driven, and the neoplasm which has followed is markedly chemo‐ and radiosensitive. For this reason, we have treated the patient with the aim of curing him even if he has a metastatic disease, choosing to avoid palliative cetuximab‐based polychemotherapy, in favor of sequential chemoradiotherapy. As a result, patient obtained a complete response yet after induction chemotherapy and we have consolidated that response performing a concurrent chemoradiotherapy protocol.

Ongoing clinical trials are evaluating the possibility to select the patients with the aim to assess their chemo‐ and radiosensitivity. Some reports support the use of a conservative treatment in HPV‐related malignancies, given their sensitivity to chemotherapy and radiotherapy.

## Conclusion

Metastatic head and neck carcinomas are often associated with poor prognosis, and the standard therapy is the palliative chemotherapy, which is associated with a median overall survival of 12 months in clinical trials. The above‐mentioned case report might be an example of the best application of translational research in oncology, namely the pretherapy study of the specific tumor‐associated “signature” and the employment of a well‐shaped therapy conformed to this “signature.” Lately, a well‐defined entity, namely HPV‐associated head and neck carcinomas, has been identified, and patients affected by HPV‐related tumors show a good chemo‐ and radiosensitivity. In the future, we can try to cure these patients albeit metastatic, using chemo‐ and radiotherapy in the right sequence.

## Consent Statement

Written informed consent was obtained from the patient for publication of this case report and any accompanying images. A copy of the written consent is available for review with the Editor‐in‐Chief of this journal.

## Informed Consent Statement

The patient provided us a written informed consent.

## Authorship

FP, GDVS, VL, and FL: collected the clinical data and reviewed the inherent literature. RA, CB, FC, and RS: reviewed the inherent literature. FP: wrote the manuscript. FF: edited the manuscript. All authors: approved the final version of the manuscript.

## Conflict of Interest

Each author declared no conflict of interests.
